# *In silico* functional analysis of the human, chimpanzee, and gorilla MHC-A repertoires

**DOI:** 10.1007/s00251-024-01369-1

**Published:** 2025-01-17

**Authors:** Griffin Kutler Dodd, Can Keşmir

**Affiliations:** Theoretical Biology and Bioinformatics, Department of Biology, Faculty of Science, Utrecht University, Utrecht, The Netherlands

**Keywords:** MHC polymorphism, Evolution, Bioinformatics, Primates, Selective sweep

## Abstract

T cells recognize peptides displayed on the surface of cells on MHC molecules. Genetic variation in MHC genes alters their peptide-binding repertoire and thus influences the potential immune response generated against pathogens. Both gorillas and chimpanzees show reduced diversity at their MHC class I A (MHC-A) locus compared to humans, which has been suggested to be the result of a pathogen-mediated selective sweep. More specifically, gorillas lack A3 lineage alleles while chimpanzees seem to have lost the A2 lineage. While previous studies showed this using phylogenetic analysis, here, we take an in silico functional approach and use the peptide-MHC binding prediction software *NetMHCpan* to examine the peptide-binding repertoires of common human, chimpanzee, and gorilla MHC-A molecules. We find that both gorillas and chimpanzees lack the A02 peptide binding specificity (supertype) despite gorillas being expected to have this specificity since they kept the A2 lineage. Additionally, we show that human MHC molecules with the A02 specificity bind fewer virus-derived peptides than other MHC molecules. We also do not find differential presentation of self-peptides by the A02 supertype, making the purpose of maintaining this specificity in high frequencies in the human population unclear. Taken together, we hypothesize that poor presentation of viral peptides by A02 supertype MHC molecules could have resulted in a selective sweep in chimpanzees and/or gorillas, though we could not identify a specific virus that may have caused this sweep.

**Supplementary Information:**

The online version contains supplementary material available at 10.1007/s00251-024-01369-1.

## Introduction

During the adaptive immune response, α:β T cells recognize short antigen fragments displayed on the surface of cells by major histocompatibility complex (MHC) molecules. MHC molecules are divided into classes based on their cell expression profiles, with class I found on the surface of all nucleated cells and responsible for presenting peptides to cytotoxic CD8^+^ T cells (Blum et al. 2013). Because of the requirement to recognize and present a diverse array of peptides, MHC genes are highly polymorphic, undergoing both balancing selection to maintain a high variation in the antigen-binding repertoire and positive selection in response to pathogen-induced selective pressure (Bernatchez & Landry 2003; Lighten et al. 2017; Pierini & Lenz 2018; Radwan et al. 2020). The combination of positive selective pressures and their well-characterized function in generating immune responses allows MHC genes to provide unique insights into evolutionary dynamics.

In humans, the classical MHC-I genes have three major loci: *HLA-A*, *HLA-B*, and *HLA-C*. The *HLA-A* alleles are phylogenetically grouped into two ancient lineages: HLA-A2 and HLA-A3. Orthologs to *HLA-A* genes are found in western gorillas (*Gogo-A*) and chimpanzees (*Patr-A*), with gorilla alleles clustering with the HLA-A2 lineage and chimpanzee alleles clustering with HLA-A3 (Adams & Parham 2001; Hans et al. 2017). Despite the highly polymorphic nature of *HLA* genes, these molecules can be classified into a few supertypes based on their peptide binding specificities (Sette & Sidney 1999; Sidney et al. 1996, 2008). HLA-A molecules have four main binding specificities, corresponding to the A01, A02, A03, and A24 supertypes, with some molecules having binding properties overlapping with two supertypes (e.g., A01/A03) (Sidney et al. 2008). It has been demonstrated that the human supertype classification can be extended to chimpanzees, providing a powerful framework for interspecies repertoire analysis (Bertoni et al. 1998; McKinney et al. 2000; Sidney et al. 2006).

Remarkably, the chimpanzee MHC-I repertoire shows a much lower diversity than the human repertoire, which per locus can have over 8000 alleles (Barker et al. 2023; Maibach et al. 2017). It is suggested that this has been caused by a pathogen-mediated selective sweep by simian immunodeficiency virus (SIV) (De Groot et al. 2002, 2008). Additionally, the A2 lineage is missing in the chimpanzee MHC molecules sequenced so far, though it is not known whether this is also due to a selective sweep or occurred by another mechanism. The gorilla MHC-I repertoire shows similarly low diversity, with the A3 lineage being lost in these species. It is also unknown whether the A3 lineage was lost during gorilla evolution by a similar selective sweep as in the chimpanzee population or whether it arose after the common ancestor of humans and chimpanzees diverged from gorillas (Hans et al. 2017). Thus far, no comparative analysis of the MHC-A repertoires in these three species has been performed, so the functional consequences of A2 and A3 loss in chimpanzees and gorillas, respectively, are currently unknown.

To analyze the functional differences between the human, chimpanzee, and gorilla MHC-A repertoires, we used *NetMHCpan* to generate MHC-peptide binding predictions in silico. *NetMHCpan* is an artificial neural network trained on binding affinity and eluted ligand mass spectrometry data to generate peptide binding predictions for any MHC molecule (Hoof et al. 2009; Reynisson et al. 2020). We clustered MHC molecules based on their binding repertoires from peptides derived from primate-infecting viruses, and we used these in silico predictions to understand why the A2 and A3 MHC lineages may have disappeared in chimpanzees and gorillas, respectively.

## Methods

### MHC phylogeny

It is not computationally feasible to analyze all known MHC molecules from each species, so we selected representative MHC molecules. Our final dataset included 19 *HLA-A* alleles spanning all four binding supertype specificities (A01, A02, A03, and A24) (Sidney et al. 2008), as well as six *Patr-A* alleles, four *Gogo-A* alleles, and an *HLA-B* allele as an outgroup in this study (Supplementary Table 1). Our selection of *Patr-A* and *Gogo-A* alleles was based on an established phylogeny published earlier (Hans et al. 2017). For each allele, coding DNA and protein sequences were obtained from the IPD-IMGT/HLA and IPD-MHC NHP databases (Barker et al. 2023; Maccari et al. 2017)*.* Coding DNA sequences were aligned using ClustalW (Larkin et al. 2007) with default parameters (IUB scoring matrix, gap opening penalty of 15, and gap extension penalty of 6.66), and phylogeny was reconstructed using neighbor-joining maximum composite likelihood implemented in MEGA11 with 1000 bootstrap replications (Tamura et al. 2021).

### Virus binding predictions

Following the approach of Maibach and Vigilant (2019), we obtained a dataset of primate-infecting viruses from Virus-Host DB (https://www.genome.jp/virushostdb), a database of viral genomes annotated with their hosts (Mihara et al. 2016), resulting in 1704 unique genomes with their associated protein sequences. To reduce the bias from closely related viruses, we computed pairwise sequence identities of Needleman-Wunsch global alignments for each pair of viruses using EMBOSS Needle and collapsed viruses with greater than 95% identity (Madeira et al. 2022). We then used *NetMHCpan* 4.1 to cut each viral protein into overlapping peptides of lengths 8–11 and generate binding score predictions for each peptide-MHC allele pair (Reynisson et al. 2020). We use a percentile rank threshold of 1% (i.e., a binding score in the top 1%) to determine the binders to MHC molecules (Sette et al. 1994). However, it has been shown that MHC repertories vary in size, so we recognize that a rank threshold may not accurately define a significant binding interaction for all MHC molecules (Paul et al. 2013; Reardon et al. 2021).

*NetMHCpan* only computes percentile ranks for alleles included in the training dataset, which does not include *Gogo-A* alleles. Following the approach employed by *NetMHCpan*, we constructed a reference dataset of natural peptides to compare each binding score against (Jurtz et al. 2017). 5000 random protein sequences were downloaded from the UniProt database, and all possible digestion products of lengths 8–12 amino acids were generated from each protein (The UniProt Consortium et al. 2023). Then, 25,000 unique digestion products of each length were randomly selected, and binding scores were computed for each peptide. The rank of a viral-derived peptide was defined as its percentile rank compared to these 125,000 binding scores. These computed rank scores correlated well with the built-in *NetMHCpan* ranks for *HLA* and *Patr* alleles (Supplementary Table 2), and therefore, we decided to use these percentile ranks throughout the paper.

### Functional clustering

For each pair of alleles, we defined the similarity in their peptide-binding repertoires as the Jaccard similarity index *J* between the sets of peptides they bind. For a particular allele pair *a, b* and virus *v*, this is the ratio of the lengths of the intersection and union of the sets of unique viral peptides bound by each allele:

1 $${J}_{ab,v}=\frac{|{a}_{v} \cap { b}_{v}|}{|{a}_{v} \cup { b}_{v}|}$$

We defined the functional distance *D* between an allele pair by averaging the Jaccard similarities for each virus and converting similarity to distance:

2 $${D}_{ab}=1-\frac{1}{N}{\sum }_{i=1}^{N}{J}_{ab,i}$$

where *N* is the number of viruses. Pairwise distances were used to cluster alleles using UPGMA implemented in the R package ape (version 7.4.1) (Paradis & Schliep 2019). Confidence in each node was estimated using 1000 bootstrap replications with 20% of the viruses using the extended majority rule consensus algorithm in PHYLIP (version 3.695) (Felsenstein 2005).

### Epitope densities

We defined an epitope density score for each virus-allele pair as the ratio of the number of viral proteins bound by that MHC allele to the number of binders expected by random chance; because we used a 1% rank threshold, the expected number of binders was 1% of the total number of digestion products. We repeated the epitope density analysis specifically on simian immunodeficiency virus by downloading all SIV sequences from NCBI virus (https://www.ncbi.nlm.nih.gov/labs/virus/vssi/) with a complete nucleotide sequence and annotated as infecting gorillas or chimpanzees (*N* = 30). Collapsing sequences with greater than 95% sequence identity resulted in 22 SIV sequences (Hatcher et al. 2017). We translated the coding DNA sequences and analyzed them as described above.

### Human proteome presentation

We obtained the human proteome from the UniProt database, giving a total of 82,493 protein sequences (The UniProt Consortium et al. 2023). We generated binding predictions for each protein as described above and compared the expected number of binders (1% of the digestion products per protein) to the observed number of binders with a 1% rank threshold.

### Statistics and plotting

Statistical analysis was performed with the SciPy Python package (Virtanen et al. 2020). Trees were visualized using Interactive Tree of Life (Letunic & Bork 2024), and other figures were generated using the Matplotlib Python package (Hunter 2007).

## Results

**Fig. 1:**
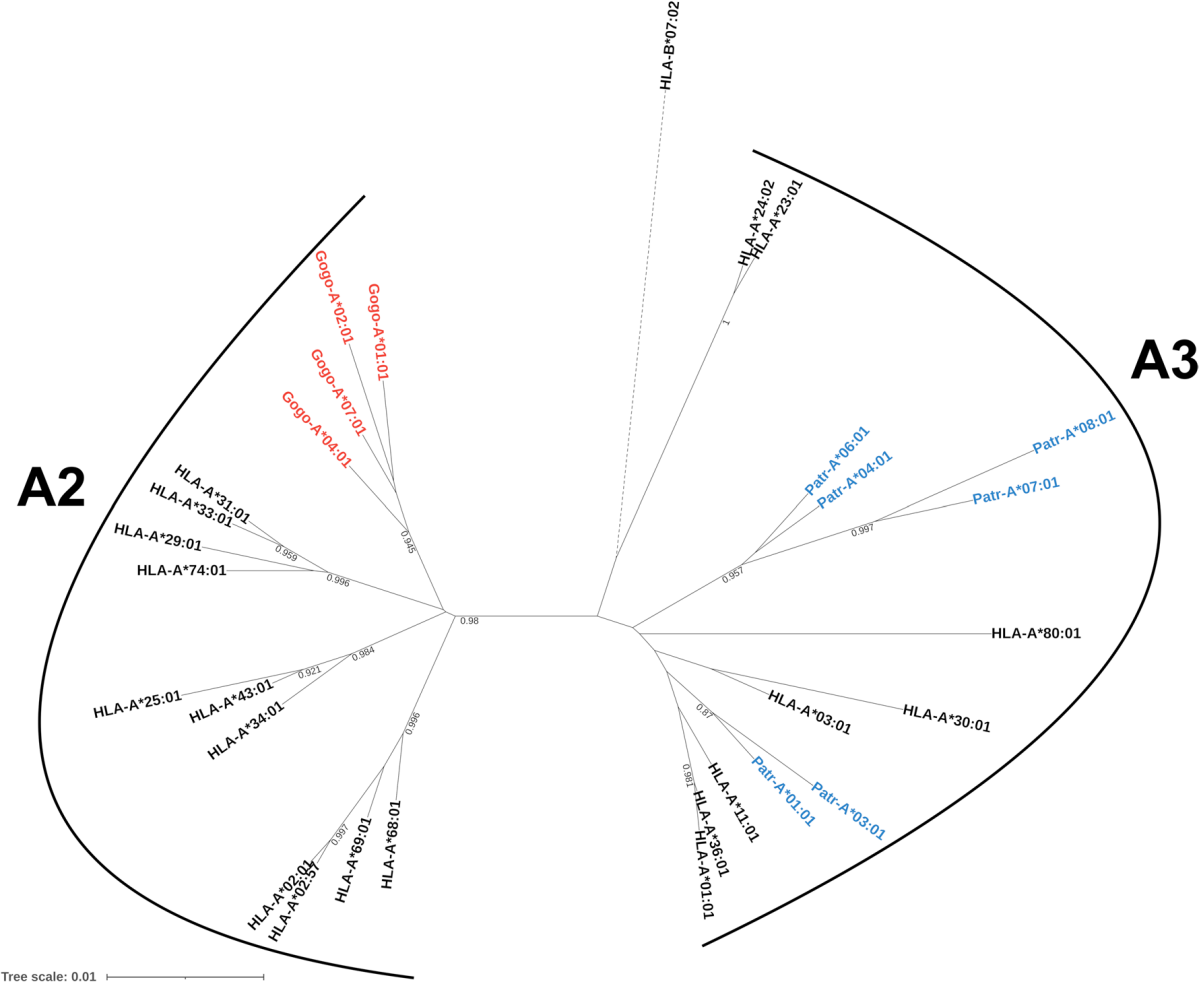
Neighbor-joining phylogenetic tree of full-length *MHC-A* exon sequences from humans (*HLA*), chimpanzees (*Patr*), and gorillas (*Gogo*), with an *HLA-B* allele as an outgroup. Gorilla and chimpanzee alleles are shown in red and blue, respectively. The branch of HLA-B*07:02 (dashed line) is shortened by a factor of 3. Bootstrap support from 1000 replicates with values greater than 0.8 are shown

To resolve the phylogenetic relationship between human, chimpanzee, and gorilla *MHC-A* alleles, we aligned exon sequences from a representative selection of alleles and created a neighbor-joining tree (Fig. 1). As has been previously reported, *HLA-A* alleles form two distinct clusters corresponding to the ancestral A2 and A3 lineages, with gorilla alleles segregating with HLA-A2 and chimpanzee alleles with HLA-A3 (Lawlor et al. 1990, 1991). This finding supports the loss of the A2 lineage in chimpanzees and either the absence or loss of the A3 lineage in gorillas (Hans et al. 2017). Additionally, we annotated each *HLA* allele with its binding specificity in Figure S1 using different colors. This reveals that all four main binding specificities (A01, A02, A03, and A24 supertypes) are represented in the A2 lineage, while the A3 lineage is missing the A02 binding specificity (supertype). The absence of the A02 supertype has been shown in a previous analysis of *Patr-A* alleles and was suggested to correspond to a possible hole in the chimpanzee repertoire (Van Deutekom et al. 2011).

**Fig. 2:**
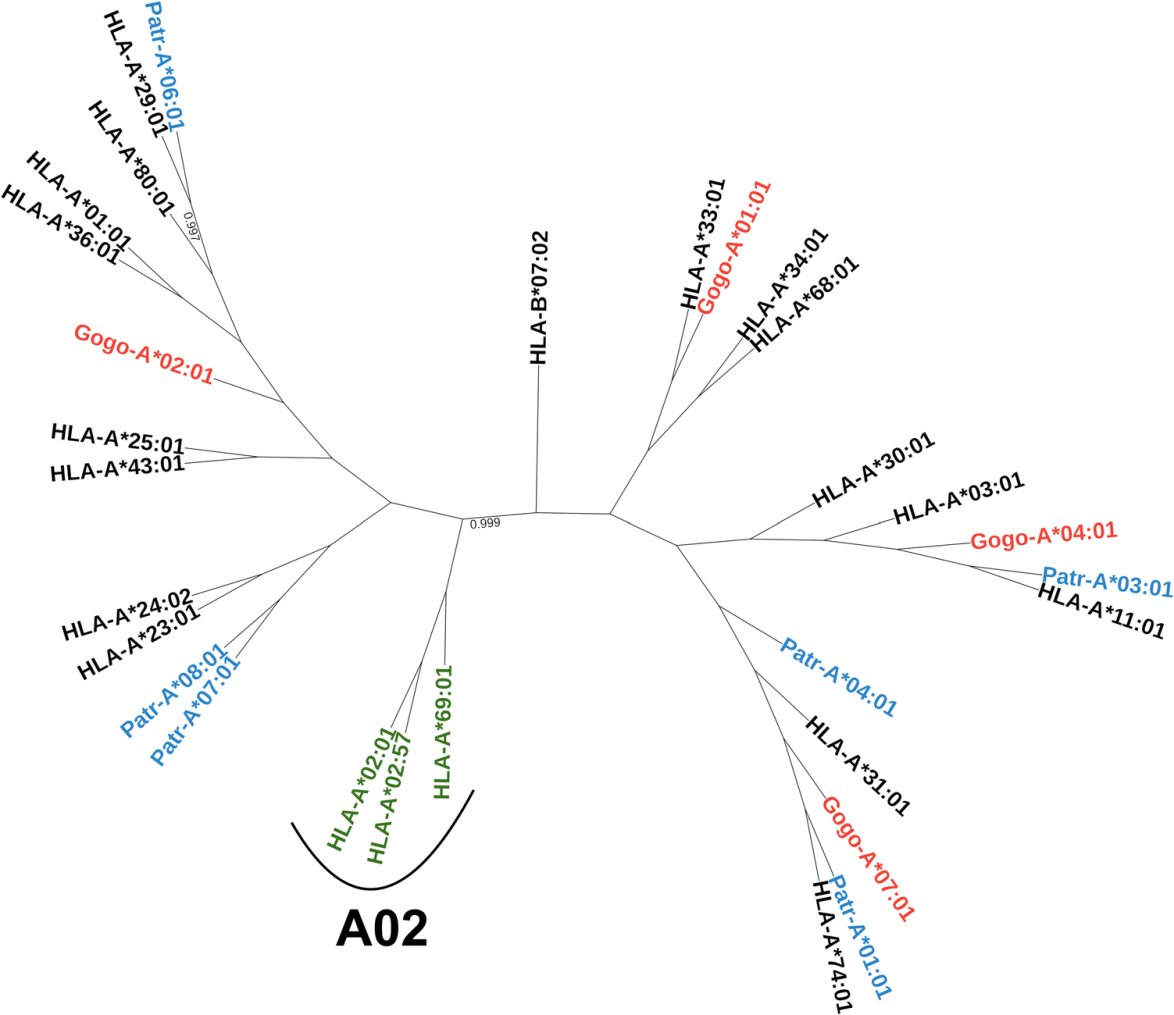
Functional clustering of MHC alleles based on the Jaccard similarities of their peptide binding repertoires. Gorilla and chimpanzee alleles are shown in red and blue, respectively. A02 supertype *HLA* alleles are shown in green, and all other *HLA* alleles are in black. All bootstrap values less than 1 are shown

Contrary to Patr-A molecules, gorillas lack MHC molecules from the A3 lineage (Fig. 1). However, the functional consequences of this are unclear: does the gorilla repertoire actually contain all four supertypes (i.e. binding specificities) included in the A2 lineage? Performing large-scale peptide-MHC binding experiments with chimpanzee and gorilla MHC molecules is very time- and material-consuming, so we have chosen to perform this analysis in silico by using *NetMHCpan* to generate binding predictions on a dataset of primate-infecting viruses (see “Methods” for the details of this computational approach). We computed a pairwise distance metric between MHC molecules based on the Jaccard similarity of their peptide repertoires. This distance metric effectively clusters *HLA-A* alleles based on their supertypes, with *Patr* and *Gogo* alleles clustering with alleles spanning the A01, A03, and A24 supertypes (Fig. 2). To ensure the observed clustering was not being driven by sequences belonging to very similar viruses, we progressively reduced the size of the virus dataset by reducing the identity threshold for collapsing redundant viruses. *HLA* alleles with the binding specificity belonging to the A02 supertype still clustered away from all *Patr* and *Gogo* alleles when viruses with more than 50% sequence identity were collapsed (Fig. S2). Thus, both primate species appear to lack A-lineage alleles with the A02 binding specificity. These results are highly unexpected, as gorillas would have been expected to have this binding specificity as they have several orthologs to the HLA-A2 lineage (Van Deutekom et al. 2011).

**Fig. 3:**
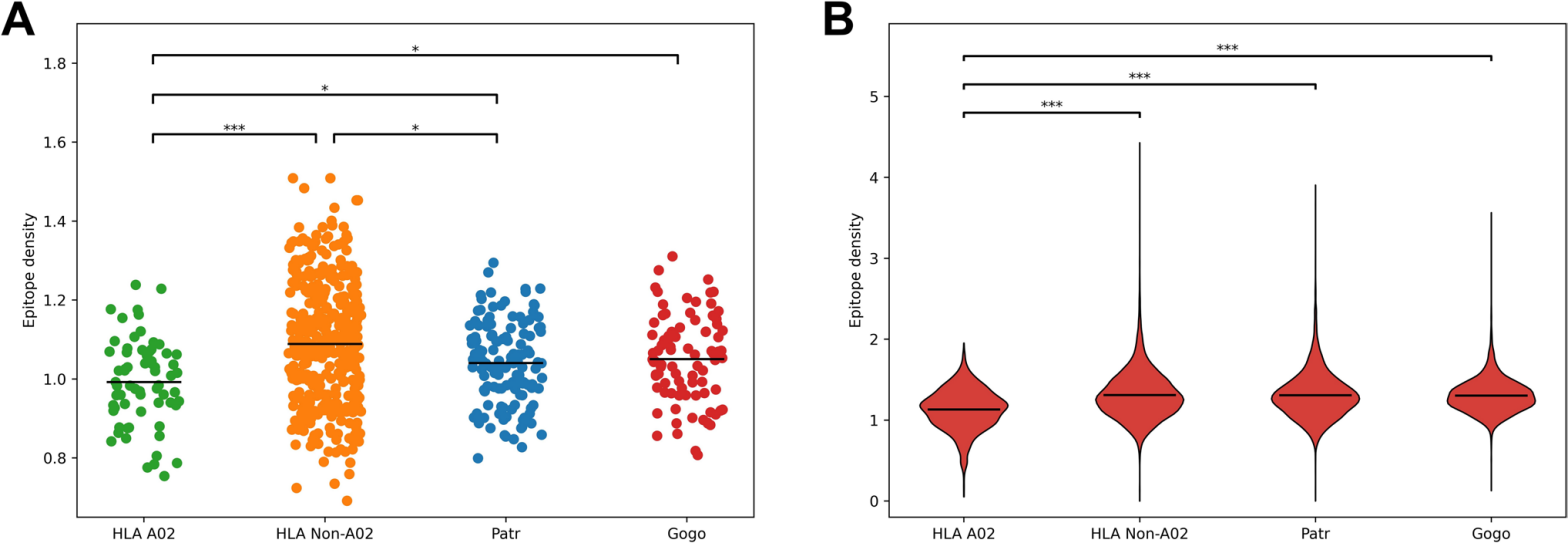
Epitope densities for simian immunodeficiency virus (**A**) and primate-infecting viruses (**B**) for A02 supertype *HLA*, non-A02 *HLA*, chimpanzee (*Patr*), and gorilla (*Gogo*) alleles. Pairwise comparisons are done with the Mann–Whitney *U* test with the Bonferroni correction. ^*^p < 0.05, ^***^p < 0.001

SIV has been proposed as being responsible for the selective sweep in the chimpanzee MHC repertoire. Therefore, we analyzed the epitope densities of several strains of SIV across MHC molecules (De Groot et al. 2002, 2008). We found that A02 supertype *HLA* alleles had a significantly lower epitope density than *HLA* molecules belonging to other supertypes, as well as gorilla and chimpanzee MHC molecules (Fig. 3A), suggesting the A02 binding specificity is poor at presenting SIV. MHC-I presentation of Gag-derived epitopes has been shown to be important for protection against HIV/SIV infection (Edwards et al. 2002; Nomura et al. 2012; Zuñiga et al. 2006). However, the A02 specificity had the highest mean epitope density for Gag protein (Fig. S3). Therefore, we do not find compelling evidence that an SIV-mediated selective sweep could have caused the loss of the A02 specificity. We then looked at epitope densities across the entire virus dataset and found that A02 alleles had a significantly lower mean epitope density (Fig. 3B), suggesting they present in general fewer viral peptides overall than other MHC molecules included in this analysis.

How can an MHC molecule that presents significantly fewer viral peptides than others become a dominant molecule globally (the frequency of HLA-A*02:01 worldwide is 24%) (Hurley et al. 2020)? One possible explanation for the widespread presence of this molecule might lie in the presentation of self-peptides. Košmrlj et al. (2010) suggested that MHC molecules presenting fewer self-peptides might result in a larger T cell repertoire (due to decreased negative selection), thereby having an increased potential to generate immune responses to pathogens. To test this hypothesis, we examined the presentation of self-antigens by the HLA-A*02:01 molecule. However, the self-presentation by the common human A02 allele HLA-A*02:01 was comparable to that of a non-A02 allele HLA-A*03:01 (Fig. S4).

Next, we wanted to identify a virus that could have caused such a selective sweep in chimpanzees and gorillas. To this end, we searched for primate-infecting viruses poorly presented by the A02 specificity and well-presented by other alleles. We filtered viruses that had a mean epitope density of less than one for all three A02 supertype molecules and a mean epitope density greater than two for at least ten non-A02 molecules. Three viruses met these criteria: Gemycircularvirus C1c, Indian encephalitis-associated cyclovirus, and Torque teno midivirus 4. However, all three of these viruses are annotated as infecting humans, making it unlikely that they caused a selective sweep in gorillas. Moreover, none of the three viruses were significantly lower presented by the A02 supertype (permutation test, results not shown).

## Discussion

Genetic analysis has shown that chimpanzees and gorillas have low nucleotide diversity at their MHC-I loci, consistent with a selective sweep reducing their MHC-I repertoires (Hans et al. 2017; Maibach & Vigilant 2019). In this study, we used bioinformatics tools to analyze the antigen presentation by MHC-A molecules in humans, chimpanzees, and gorillas. To this end, we generated in silico binding predictions on a dataset of primate-infecting viruses and used these predictions to cluster MHC molecules based on their peptide repertoires. Interestingly, we could not identify any gorilla MHC molecules having the A02 binding specificity despite the gorilla alleles included in our analysis being part of the ancestral A2 lineage. Chimpanzee alleles also lack the A02 binding specificity, a more expected result as the A2 lineage is shown to be absent in chimpanzees (Adams & Parham 2001). Obviously, we could not include all chimpanzee and gorilla MHC alleles found (or to be found) in the population in our analysis. Therefore, our analysis suggests that the A2 specificity is missing among chimpanzee and gorilla MHC molecules closely related to human A2 molecules. In other words, we cannot claim that the A02 specificity is completely absent in these species; however, it is absent in the molecules where it is most expected. These results demonstrate that functional analysis can characterize relationships that would not have been predicted from the phylogeny of the alleles.

Chimpanzees naturally infected with SIV typically do not develop symptoms and often show a strong CTL response towards conserved HIV-1 epitopes (Balla-Jhagjhoorsingh et al. 1999; Sharp et al. 2005). Particularly, MHC molecules with A03-like binding preferences, which are highly frequent in chimpanzee populations, strongly present several conserved epitopes (De Groot et al. 2020). Indeed, we observed functional clustering of several chimpanzee and gorilla MHC-A molecules with A03 supertype HLA molecules (Fig. 2), while SIV epitopes were poorly presented by the A02 specificity (Fig. 3). Can these observations explain the absence of this supertype in chimpanzees and gorillas? On average, A02 HLA molecules presented fewer viral peptides compared to other MHC binding specificities (Fig. 3). Moreover, HLA-A02 molecules had the highest presentation of Gag-derived peptides (Fig. S3), a property often reported as protective in human HIV infection (Kiepiela et al. 2007). Thus, our results do not provide compelling evidence for SIV causing a selective sweep leading to the loss of the A02 specificity, in line with reports that SIV primarily caused positive selection at the chimpanzee *Patr-B* locus (De Groot et al. 2008). However, our approach only allowed us to look at the average presentation of viral peptides, but the protective effects of HLA in controlling HIV infection are often due to the presentation of specific epitopes with high immunogenicity (Matthews et al. 2012; Pereyra et al. 2014). Additionally, no *Gogo-A* alleles are included in the training dataset for *NetMHCpan*, so the accuracy of these predictions may be lower than the predictions for humans and chimpanzees.

While we do find globally low peptide presentation by A02 supertype MHC molecules, which may have resulted in the loss of this binding specificity due to a lack of positive selection, there are other explanations for our results. For example, it is known that there is strong linkage disequilibrium between *HLA* genes. While linkage is usually strongest between the B- and C-loci, there are several common haplotypes where A- and B-locus genes are linked, such as human A1-B8 (Dawkins et al. 1999; Worwood et al. 1997). It is possible that A2-lineage genes were not in linkage with *Patr-B* genes positively selected during a selective sweep, leading to the loss of the A2 lineage in chimpanzees. However, it is surprising that gorillas would also lack the A02 specificity despite having modern orthologs to the A2 lineage. For this reason, we believe selection based on functionality is a more likely explanation than linkage disequilibrium to explain the loss of the A02 specificity in both species.

A02 supertype alleles, particularly the HLA-A*02 group, are extremely abundant worldwide (Arrieta-Bolaños et al. 2023). The dominant HLA-A*02 allele differs with ethnicity (Chen et al. 2012), suggesting that the abundance of the A2 group is not due to a founder effect but rather many independent selection events. It is thus surprising that they appear to be less efficient at presenting viral peptides in our analysis. Calis et al. (2010) showed that most human and chimpanzee MHC-A molecules are biased towards presenting low G + C content peptides, which appear to be overrepresented in pathogens. Interestingly, HLA-A*02:01 was shown to be uncorrelated to G + C content in their analysis, suggesting a mechanism for the lower presentation of viral peptides by the A02 specificity. However, we could not determine why this specificity is present in high frequencies in the human population, especially as we did not see evidence of lower self-presentation by HLA-A*02:01 (Fig. S4). Thus, further analysis is needed to determine the reason why humans retained the A02 specificity while chimpanzees and gorillas did not.

## Supplementary Information

Below is the link to the electronic supplementary material.

Supplementary file1 (PDF 1955 KB)

## Data Availability

The dataset analyzed during this study can be found in the MHC-A-functional-analysis repository on GitHub, https://github.com/gkutlerdodd/MHC-A-functional-analysis/.
