# Supplementary material for: In silico functional analysis of the human, chimpanzee, and gorilla MHC-A repertoires
Source: Immunogenetics. 2025 Jan 17;77(1):12. doi: 10.1007/s00251-024-01369-1 (PMC11741996; doi:10.1007/s00251-024-01369-1)
Supplement: Supplementary file 1 — Supplementary file1 (PDF 1955 KB) [file 251_2024_1369_MOESM1_ESM.pdf]

Title: Functional Analysis of the Human, Chimpanzee, and Gorilla MHC-A Repertoires

Journal: Immunogenetics

Authors: Griffin Kutler Dodd[1], Can Keşmir[1]

Affiliations:

[1]Theoretical Biology and Bioinformatics, Department of Biology, Faculty of Science, Utrecht University, Utrecht, The Netherlands

Corresponding author: Can Keşmir

E-mail: c.kesmir@uu.nl

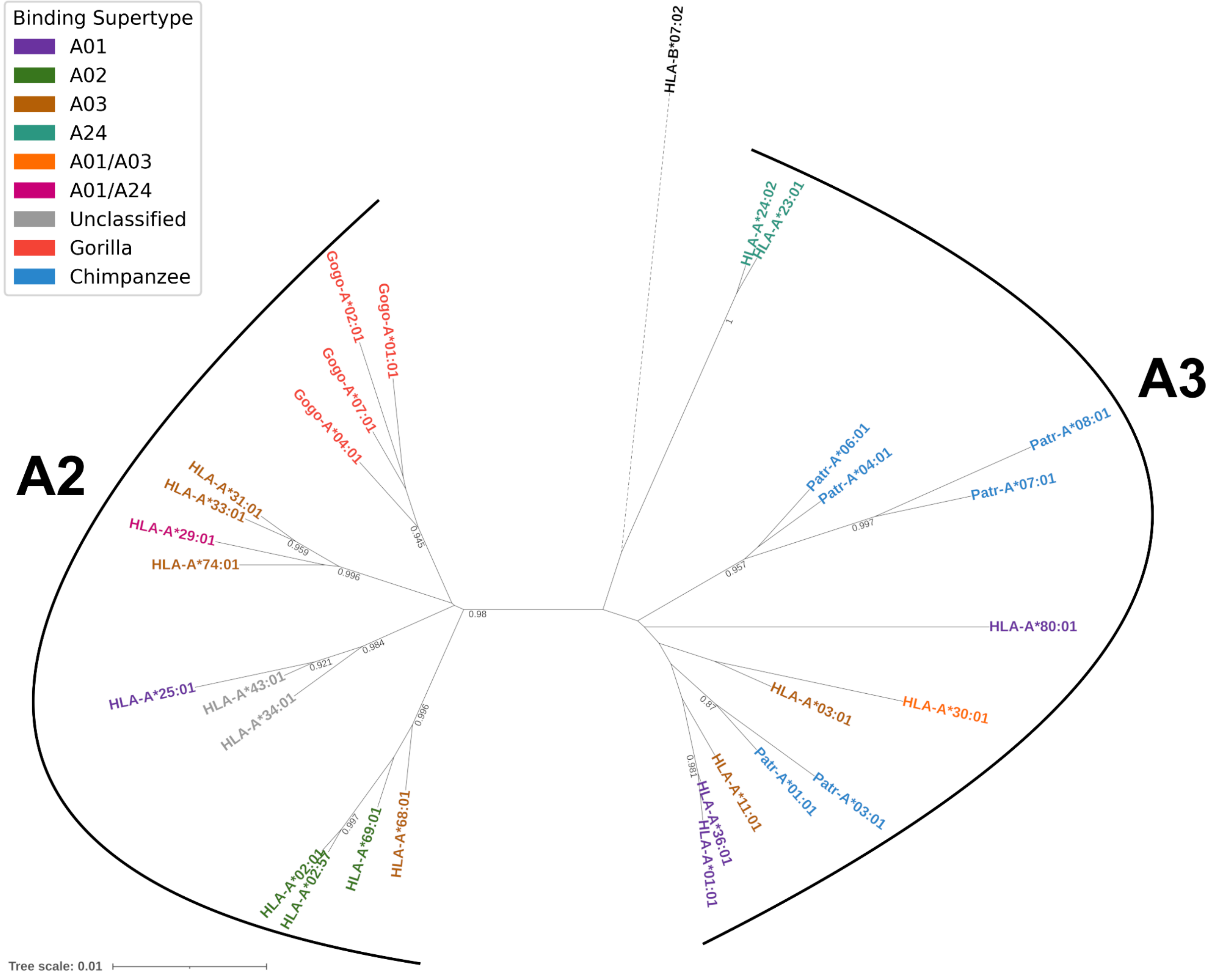

Supplementary Figure 1. Neighbor-joining phylogenetic tree of full-length *MHC-A* exon sequences from humans (*HLA*), chimpanzees (*Patr*), and gorillas (*Gogo*), with an *HLA-B* allele as an outgroup. Binding supertypes of the HLA-A alleles are shown in the figure legend. The branch of *HLA-B*07:02* (dashed line) is shortened by a factor of 3. Bootstrap support from 1000 replicates with values greater than 0.8 are shown.

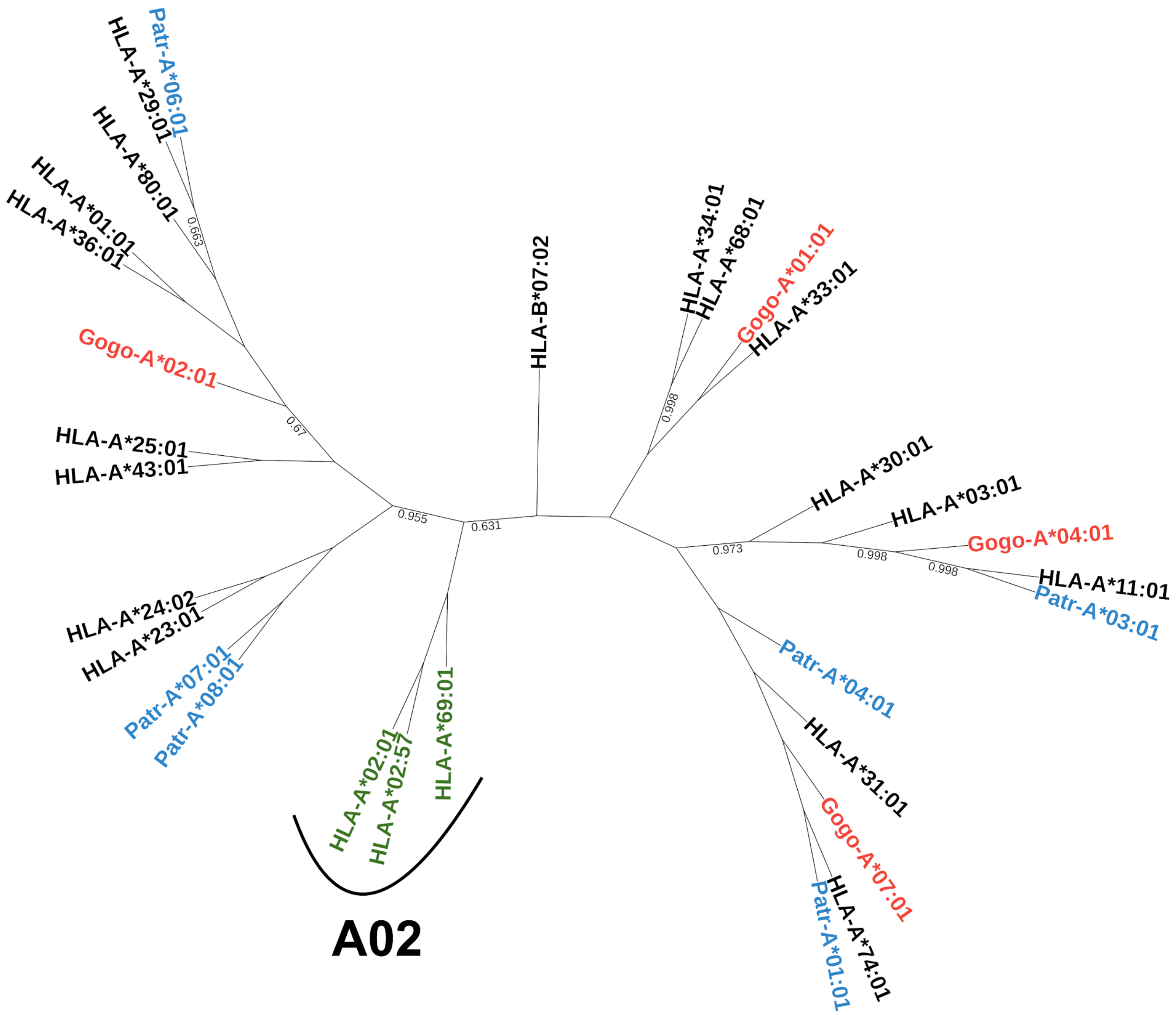

Supplementary Figure 2. Functional clustering of MHC alleles based on the Jaccard similarities of their peptide binding repertoires using a 50% identity threshold for virus inclusion. Gorilla and chimpanzee alleles are shown in red and blue, respectively. A02 supertype *HLA* alleles are shown in green, and all other *HLA* alleles are in black. All bootstrap values less than 1 are shown. HLA-B*07:02 is used as an outgroup.

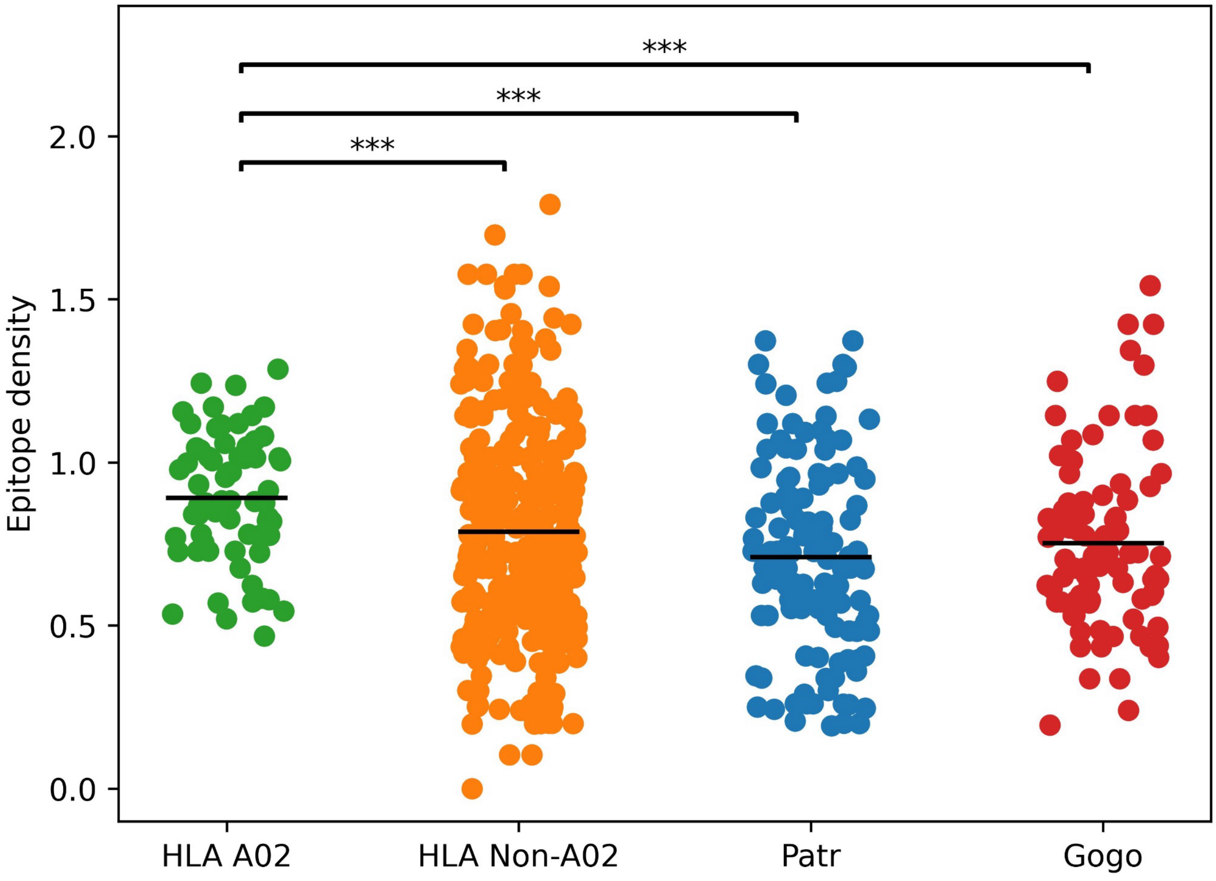

Supplementary Figure 3. Epitope densities of SIV Gag proteins in A02 supertype *HLA*, non-A02 *HLA-A*, chimpanzee (*Patr*), and gorilla (*Gogo*) alleles. Pairwise comparisons are done with the Mann-Whitney U test with the Bonferroni correction. *** = $p < 0.001$.

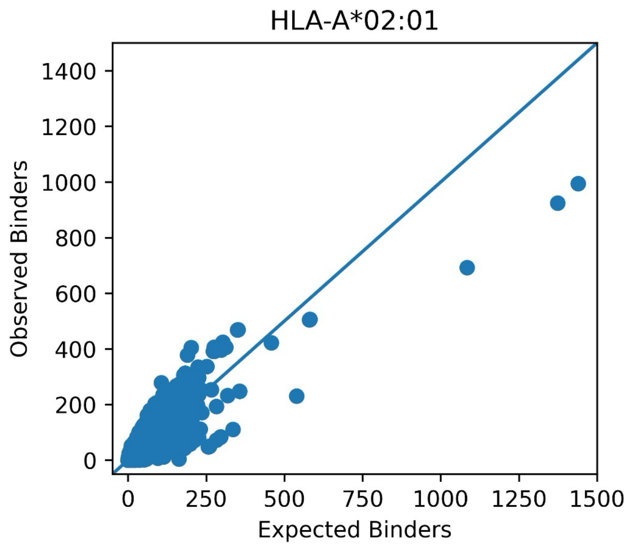

Supplementary Figure 4. Self-presentation of the human proteome by *HLA-A*02:01* and *HLA-A*03:01*. Each point represents the peptides derived from one protein that are strong binders to that MHC molecule, as well as the number that would be expected to bind by random chance treating the top 1% of binding interactions as strong. Proteins below the line have fewer strong-binding peptides than expected, while proteins below the line have more.

Supplementary Table 1. MHC alleles included in this study and their binding supertypes if applicable. All alleles refer to the first variant of the lineage (e.g. *Gogo-A*01:01* refers to *Gogo-A*01:01:01*).

Allele	HLA-A Supertype
*HLA-A*01:01*	A01
*HLA-A*02:01*	A02
*HLA-A*02:57*	A02
*HLA-A*03:01*	A03
*HLA-A*11:01*	A03
*HLA-A*23:01*	A24
*HLA-A*24:02*	A24
*HLA-A*25:01*	A01
*HLA-A*29:01*	A01/A24
*HLA-A*30:01*	A01/A03
*HLA-A*31:01*	A03
*HLA-A*33:01*	A03
*HLA-A*34:01*	Unclassified
*HLA-A*36:01*	A01
*HLA-A*43:01*	Unclassified
*HLA-A*68:01*	A03
*HLA-A*69:01*	A02
*HLA-A*74:01*	A03
*HLA-A*80:01*	A01
*HLA-B*07:02*	N/A
*Patr-A*01:01*	N/A
*Patr-A*03:01*	N/A
*Patr-A*04:01*	N/A
*Patr-A*06:01*	N/A
*Patr-A*07:01*	N/A
*Patr-A*08:01*	N/A
*Gogo-A*01:01*	N/A
*Gogo-A*02:01*	N/A
*Gogo-A*04:01*	N/A

*Gogo-A*07:01*	N/A

Supplementary Table 2. Pearson correlation between built-in NetMHCpan binding rank and computed rank scores using 50 random viruses for all human (*HLA*) and chimpanzee (*Patr*) alleles.

**Allele**	**Correlation**	**p-value**
*HLA-A*01:01*	1.0	1.7e-07
*HLA-A*02:01*	1.0	5.2e-08
*HLA-A*02:57*	1.0	3.2e-07
*HLA-A*03:01*	1.0	9.0e-08
*HLA-A*11:01*	1.0	2.7e-07
*HLA-A*23:01*	1.0	3.0e-06
*HLA-A*24:02*	1.0	1.0e-04
*HLA-A*25:01*	1.0	8.5e-07
*HLA-A*29:01*	1.0	1.8e-05
*HLA-A*30:01*	1.0	2.0e-08
*HLA-A*31:01*	1.0	1.1e-06
*HLA-A*33:01*	1.0	7.2e-08
*HLA-A*34:01*	1.0	2.0e-08
*HLA-A*36:01*	1.0	1.3e-08
*HLA-A*43:01*	1.0	2.4e-08
*HLA-A*68:01*	1.0	3.3e-08
*HLA-A*69:01*	1.0	5.0e-09
*HLA-A*74:01*	1.0	3.4e-06
*HLA-A*80:01*	1.0	7.9e-07
*HLA-B*07:02*	1.0	9.6e-06
*Patr-A*01:01*	1.0	2.4e-05
*Patr-A*03:01*	1.0	4.7e-07
*Patr-A*04:01*	1.0	1.8e-04
*Patr-A*06:01*	1.0	2.6e-09
*Patr-A*07:01*	1.0	2.0e-06
*Patr-A*08:01*	1.0	6.9e-07
